# Expert perspectives on tuberculosis screening procedures for migrants

**DOI:** 10.36416/1806-3756/e20240347

**Published:** 2024-12-17

**Authors:** Marina Pinheiro, David N Moreira, Ana Aguiar, Raquel Duarte

**Affiliations:** 1. Unidade Local de Saúde de Barcelos/Esposende, EPE, Barcelos/Esposende, Portugal; 2. Departamento de Ciências Químicas, Faculdade de Farmácia, Universidade do Porto, Porto, Portugal; 3. EPIUnit ITR, Instituto de Saúde Pública da Universidade do Porto, Universidade do Porto, Porto, Portugal.; 4. Estudos das Populações - Instituto de Ciências Biomédicas Abel Salazar, Universidade do Porto, Porto, Portugal; 5. Instituto de Saúde Pública Doutor Ricardo Jorge - INSA Porto - Porto, Portugal

**Keywords:** Europe, Communicable diseases, Transients and migrants, Public health, Tuberculosis

## Abstract

**Objective:**

To evaluate the perspectives of tuberculosis experts from different countries regarding national screening procedures.

**Methods:**

This was a qualitative descriptive study. Data were collected by using electronic, anonymized surveys with experts in tuberculosis in seven different countries within two World Health Organization regions (Europe and Africa). Thematic analysis was employed.

**Results:**

The survey results indicate that there are varied perceptions of and experiences with national guidelines on screening for and treatment of tuberculosis (especially in the population tested), the appropriate timing of screening, types of tests, best practices, barriers, and limitations of the screening. The participants highlighted the importance of integrating health care services into the community to achieve people-centered health care. The study also sheds light on the importance of involving trained nurses and social workers in the screening process and of networks to ensure continuity of care.

**Conclusions:**

The overall perceptions of the respondents underscore the importance of standardized screening guidelines. The ongoing collaboration between public health services, the private sector, and the community is essential to reduce tuberculosis transmission, as well as to provide substantial public health and economic benefits.

## INTRODUCTION

Tuberculosis continues to be the leading cause of death from a single infectious disease agent among adults worldwide.^1^ In 2022, tuberculosis was responsible for 1.3 million deaths and accounted for a mean of 1.85% (range, 1.69-2.05%) of disability-adjusted life-years (DALYs) worldwide across all causes of disease, regardless of age and sex. In 2021, in the World Health Organization (WHO) European region, tuberculosis accounted for a mean of 0.16% (range, 0.15-0.17%) of the total deaths and a mean of 0.2% (range, 0.18-0.2%) of DALYs worldwide.^2^ Controlling tuberculosis in the migrant community is highly complex because the extraordinary magnitude of migration patterns has created significant obstacles to the local control of infectious diseases.^3^ Migration has increased worldwide, with a particularly marked impact in European countries, and is one of the most pressing public health challenges of our time.^4^ ^-^ ^6^ Migrants account for 5.1% of the population of Europe, which now includes an estimated 23 million people from non-European Union countries.^7^ In several European countries, migrants account for the majority of patients with tuberculosis. Evidence from the WHO and European Centre for Disease Prevention and Control (ECDC) supports screening all migrants for active tuberculosis with chest X-rays and screening immigrants from high tuberculosis-burden countries for latent tuberculosis infection (LTBI) by using tuberculin skin tests (TSTs) or interferon-gamma release assays (IGRAs).^8^ ^-^ ^11^ Differences in screening protocols across Europe underscore the need for standardized practices. The aim of this study was to review screening guidelines, gathering insights on national protocols from tuberculosis experts in various countries, to develop recommendations for a Delphi consensus statement on harmonizing tuberculosis screening practices.

## METHODS

This study used qualitative methods, including an online survey with open and closed questions, targeting experts from seven countries in the WHO regions of Europe and Africa. The experts, all of whom were individuals responsible for tuberculosis patient care or national guideline influence, included clinicians, public health professionals, researchers, and industry members. The survey aimed to capture expert opinions on a broad range of subjects, in order to achieve theoretical saturation. Distributed via Microsoft Forms, the survey was emailed to experts through the International Union Against Tuberculosis and Lung Disease (IUATLD), a global technical and scientific organization.

The survey questions covered participant demographics (sex, age, and level of education), geographic region, country of practice, sector of activity, years of experience in the area of tuberculosis, involvement with tuberculosis in migrants, direct patient contact, and the annual number of tuberculosis cases managed. Other questions elicited the perspective of the experts on the following topics: at what point screening for latent and active tuberculosis should occur; who is responsible for identifying the cases that should undergo tuberculosis screening; what tests should be recommended for screening for latent and active tuberculosis; what kind of support migrants with latent and active tuberculosis should receive in terms of treatment, continuity of care, and follow-up; the recommended practices for tuberculosis screening in migrant populations; and the barriers to, limitations of, and potential harms in screening for tuberculosis in the migrant population. The IUATLD secretariat invited members via email, and participants consented, with identifying data accessible only to the research team. The data were collected between March and May of 2024 and were exported to an Excel spreadsheet. Employing an inductive approach, we developed a thematic analysis framework to categorize themes, subthemes, and emerging categories from participant answers to the open-ended answers. The thematic analysis was manually conducted using a Word document according to the protocol devised by Braun and Clarke.^12^ The lead investigator conducted the initial coding and discussed it with the rest of the team, subsequently making adjustments. To guarantee trustworthiness, we followed the thematic analysis phases proposed by Nowell et al*.* ^13^:

1. “Becoming familiar with the data”-thoroughly reading and re-reading all statements, taking notes on possible themes, and organizing the data in a table format using a word processor
2. “Creating initial codes”-utilizing a codebook specially developed for this study
3. “Identifying themes”-ensuring, in an inductive (bottom-up) manner, that the themes were closely tied to the native language of the participant (This data-driven approach involved coding the data without forcing it into a pre-existing coding framework.)
4. “Reviewing themes”-one researcher conducting the initial coding and another reviewing and refining the codes related to the identified themes, until a consensus is achieved
5. “Defining and naming themes”-designating the themes in the most self-explanatory way possible
6. “Writing the report”-reviewing and validating the themes for additional member checking and production of the final codes and themes. The selected quotes were translated hermeneutically to enhance clarity without changing their meaning.
7. The study was approved by the Ethics Committee of the University of Porto Institute of Public Health, in the city of Porto, Portugal (Reference no. CE24260). All stages of the study were conducted in accordance with the ethical guidelines of the Declaration of Helsinki. Study documents were securely stored and encrypted with password protection.

## RESULTS

The study participants (respondents) comprised eleven experts, most (72.7%) of whom were men, with a mean age of 55.9 ± 9.4 years and a mean number of years of experience in the area of tuberculosis of 27.5 ± 9.7. Nearly all (90.9%) had experience with migrant populations. Among those directly treating patients (n = 5), the mean annual number of tuberculosis cases managed was 160 ± 116. Over half (54.5%) of the eleven respondents had tuberculosis screening experience. The respondents were working in a total of seven countries: the United Kingdom (n = 4); Netherlands (n = 2); Germany (n = 1); Finland (n = 1); Switzerland (n = 1); Italy (n = 1); and New Zealand (n = 1). The overall characteristics of the experts are shown in Table 1.

**Table 1:** Characteristics of the study participants.^a^

Variable	(N = 11)
Sex	
Male	8 (72.7)
Female	3 (27.3)
Age (years), mean ± SD	55.9 ± 9.4
Highest academic degree	
Doctorate	7 (63.6)
Bachelor’s	2 (18.2)
High school diploma or equivalent	1 (9.1)
Other	1 (9.1)
Years of experience in tuberculosis, mean ± SD	27.5 ± 9.7
Annual number of cases managed,^b^ mean ± SD	160 ± 116
Country of practice	
United Kingdom	4 (36.3)
Netherlands	2 (18.2)
Finland	1 (9.1)
Germany	1 (9.1)
Switzerland	1 (9.1)
Italy	1 (9.1)
New Zealand	1 (9.1)
World Health Organization region	
Europe	9 (81.8)
Africa	2 (18.2)
Primary area of activity	
Academia	5 (45.4)
Public sector	4 (36.3)
Civil society	1 (9.1)
Other	1 (9.1)

a Values expressed as n (%), except where otherwise indicated. ^b^Only seven (63.6%) of the respondents had direct contact with tuberculosis patients in their daily practice.

The majority (63.6%) of the experts agreed that immigrants should be screened for tuberculosis upon entry into the host country, whereas 27.2% were of the opinion that screening should occur before entry and 27.2% were of the opinion that it should occur after entry. According to 54.5% of the experts, in their countries, public health services are primarily responsible for identifying cases that should undergo tuberculosis screening. Three respondents (27.2%) reported that screening occurs at migration centers, and two (18.3%) reported that it occurs at primary care facilities. According to the experts, the main tests recommended for screening (for active tuberculosis and for LTBI) were as follows (proportion of respondents): X-ray (90.9%); IGRA (64.0%); symptom questionnaire (36.0%); QuantiFERON-TB Gold In-Tube assay (18.2%); TST (18.0%); and molecular testing (9.1%). The perceptions of the experts regarding screening are presented in Table 2.

**Table 2:** Perceptions of experts (N = 11) regarding screening migrants for tuberculosis.

Variable	n (%)
Timing	
Upon entry	7 (63.6)
Pre-entry	3 (27.2)
Post-entry	3 (27.2)
Authority responsible	
Public health	6 (54.5)
Migration services	3 (27.2)
Primary care	2 (18.3)
Tests	
X-ray	10 (90.9)
Interferon-gamma release assay	7 (64.0)
Symptoms questionnaire	4 (36.0)
QuantiFERON-TB Gold In-Tube assay	2 (18.2)
Molecular testing	1 (9.1)

The analysis of the open-ended questions identified three main themes: 1) perceptions of the support that migrants should receive; 2) perceptions about recommended practices; and 3) perceptions about barriers to, limitations of, and potential harms of screening. Respondent ages are reported as their ages at the time of their participation in the study. Table 3 displays the themes, subthemes, and categories derived from the thematic analysis. Below, we give an account of participant descriptions of the emerging themes.

**Table 3:** Themes, subthemes, and categories derived from a thematic analysis of the survey responses.

Theme	Sub-theme	Category	Definition	Sample expert quotations
Perceptions about support that migrants with latent tuberculosis and active tuberculosis should receive	Treatment	Procedure	This theme aims to describe participant understanding of the support that migrants should receive during the screening procedure, in which the national system of the host country is involved, and the coordination of that system with other services.	“Tuberculosis treatment (and preventive therapy) should be completely free of charge for the person (which is not the case in Germany).” - male, 57 years
	Continuity of care	Support	This theme aims to describe participant understanding of the continuity of care that migrants should receive during the screening procedure, in which the national system of the host country is involved, and the coordination of that system with other services.	“Offer tuberculosis screening as part of a health care package, rather than a stand-alone process, with screening and support for other conditions.” - male, 57 years
	Follow-up	Prevention	This theme aims to describe participant understanding of the follow-up that migrants should undergo during the screening procedure, in which the national system of the host country is involved, and the coordination of that system with other services.	“Free treatment and follow-up to completion of treatment (12 months after completion of treatment for drug-resistant tuberculosis); If preventive treatment declined, follow-up for 2 years . . .” - male 67 years
Perceptions about recommended screening practices	Perceptions about the usefulness of protocols	Procedures	This theme aims to describe participant knowledge about national and international screening protocols.	“Current practices are not based on the recent literature in all countries. The practices inside countries might also vary. It would be very useful to have common renewed guidelines for European low incidence countries or probably also other European Union countries would be beneficial. The same recommendations are not suitable for every country in the world.” - female, 57 years
	Insights about the protocol used	Procedures	This theme aims to describe participant priorities regarding and comprehension of their local screening protocols.	“Detecting active tuberculosis among people coming from highly endemic countries [. . .] But for the elimination of tuberculosis, all migrants need to be screened for tuberculosis infection afterwards as well.” - male, 48 years
Perceptions about barriers to screening	Potential harms	Stigma and discrimination	This theme aims to describe participant understanding of the potential harms of screening.	“Stigma, concerns that they may be deported once they access care . . .” - male, 45 years
	Barriers	Accessibility	This theme aims to describe participant knowledge about the main barriers of screening	“[. . .] direct and indirect costs of access to care.” - male, 45 years
		Accessibility		“Poor documentation and mapping of migrant population”. - male, 38 years
		Accessibility		“[. . .] no understanding of the local language and practices; direct and indirect costs of access to care; lack of trust in health care providers.” - male, 45 years
		Health literacy		“Migrants from tuberculosis-endemic nations frequently lack an understanding of latent tuberculosis infection conceptually, and it is time-consuming to incorporate this educational element into the process”. - female, 54 years
		Policy		“In Germany, there is as yet no comprehensive strategy to combat tuberculosis; quite low tuberculosis competence generally at public health care facilities, clinics, hospitals, and general practitioner offices [. . .] and money, the problem seems too small for most politicians.” - male, 57 years

### Perceptions of the support that migrants should receive

Concerning the perception of the experts regarding the support and specifically the treatment that migrants with latent or active disease should receive, almost half (45.4%) of the participants pointed out the need for access to free tuberculosis health care services and free treatment for active tuberculosis and LTBI.

“*Tuberculosis treatment (and preventive therapy) should be completely free of charge for the person (which is not the case in Germany).*”

*- Male, 57 years*

The involvement of the community was mentioned several times throughout the participant responses and was considered the key element in coordinating with the health services.

*“Migrants require an invitation to acquire a new approach to accessing health care and make optimal use of community service in the new country. Screening activities lend themselves to engagement in health care processes and developing partnership behaviors in health care. Therefore, in this setting, I would recommend providing a ‘community’ for the treatment to be administered/monitored-a local pharmacy, clinic, and dedicated general practitioner-focusing on offering more than drug therapy, making this a place of engagement in re-orientating health-related behaviors and literacy.”*

*- Male, 54 years*

Social support was also identified as fundamental during the continuity of care. The respondents consistently highlighted the importance of offering screening as part of a health care package, rather than as a stand-alone procedure, offering social support, including accommodation and food in close support tailored to the needs of the individual.

*“[. . .] stable accommodation, benefits, and food for the duration of tuberculosis treatment . . .”*

*- Male, 57 years*

The participants also pointed out that person-centered care, health education, and literacy are essential for continuity of care. Another important element considered important by the participants for the screening of tuberculosis in migrants was the use of video-observed therapy or directly observed therapy. Participants consistently emphasized the need for physicians, nurses, and social workers to play an active role during the screening procedures.

*“[. . .] video-observed therapy or directly observed therapy, social support if needed . . .”*

*- Female, 57 years*

Regarding follow-up, the participants also highlighted the need for continuous care. According to the experts, the exact follow-up time depends on clinical conditions. In contrast, some experts suggested that the follow-up should occur during the period of treatment completion; one of the participants suggested a follow-up period of 12 months after completion of treatment for drug-resistant tuberculosis and 2 years in the case of preventive treatment refusal.

*“Free treatment and follow-up to completion of treatment (12 months after completion of treatment for drug-resistant tuberculosis); if preventive treatment declined, follow-up for 2 years . . .”*

*- Male, 67 years*

From the evaluation of the participant’s perspectives, there is a consensus that the screening of tuberculosis should exclude active tuberculosis, especially from high endemic countries of tuberculosis.

*“All migrants from tuberculosis-endemic countries should be screened as per local or ECDC recommendations.”*

*- Male, 45 years*

The participants perceive that screening for infection is also crucial to eliminating tuberculosis.

*“Detecting active tuberculosis among people coming from highly endemic countries [. . .] But for the elimination of tuberculosis, all migrants need to be screened for tuberculosis infection afterwards as well.”*

*- Male, 48 years*

Some respondents emphasized that screening protocols should take into consideration the characteristics of the target population and the duration of their stay in the host country.

*“It depends on the type of migrant and the reason to migrate: symptom screening among refugees under humanitarian emergency; chest X-ray and other examinations . . .”*

*- Male, 69 years*

For instance, for short stays by individuals from tuberculosis-endemic countries, including students, temporary workers, and visitors, the screening should cover active tuberculosis using X-ray and sputum smear microscopy. Screening for active tuberculosis should be performed for all individuals who will have an extended stay (≥ 6 months). In addition, the use of an IGRA for detecting LTBI should be considered for individuals under 55 years of age.

*“I would add that transient populations (students, temporary workers, and visitors) from endemic nations require screening of at least active pulmonary tuberculosis by chest X-ray and sputum algorithm for any abnormalities. Entrants planning a longer stay (≥ 6 months) should be screened for active tuberculosis, and individuals under 55 years of age require screening for LTBI by X-ray of the chest, IGRA, and QuantiFERON.”*

*- Female, 54 years*

Entry screening practices for migrants vary by country and often include multiple stages and methods. The approaches range from mandatory screenings for active tuberculosis upon arrival to follow-up screenings for LTBI, especially for individuals from high-incidence regions or those staying for longer periods. This comprehensive approach aims to identify and manage tuberculosis infections early, thereby preventing the spread of the disease.

*“In my country, the entry screening for migrants who are coming from highly endemic countries and plan to stay in the Netherlands for > 3 months are mandatorily screened for tuberculosis. Other persons are screened with an X-ray at entry and follow-up screening for 2 years (not mandatory). We plan to replace these follow-up screenings with a tuberculosis infection screening shortly after entry (the first X-ray).”*

*- Female, 59 years*

*“Adult migrants from high- and middle-incidence countries in collective housing settings or mass accommodation should undergo X-ray screening before moving in; adult working migrants from high-incidence countries in vulnerable professional settings (e.g. hospitals, nursing homes, and kindergartens) should also undergo X-ray screening, as well as being offered LTBI screening and preventive therapy, if necessary. All adult migrants from high-incidence countries (> 100/150 cases/100,000 population?) or with a history of contact with tuberculosis should be offered LTBI screening and preventive therapy at immigration or soon thereafter. All children from middle- or high-incidence countries or who are close contacts of adults with a history of tuberculosis should undergo LTBI screening and receive preventive therapy if needed.”*

*- Male, 58 years*

One respondent considered the detection of infection too expensive to be performed *en masse*. Screening for active tuberculosis can be performed by using the symptoms questionnaire known as the MM-CHECK, which is available in 33 languages.^14^ Another respondent made the observation that the guidelines for migrant screening varied across European countries and were not based on the most recent literature. That respondent proposed the establishment of uniform guidelines for use throughout Europe.

*“Current practices are not based on the recent literature in all countries. The practices inside countries might also vary. Having common renewed guidelines for low-incidence European countries or probably other European Union countries would be beneficial. The same recommendations are not suitable for every country in the world.”*

*- Female, 57 years*

Most participants pointed out that stigma is one of the most critical barriers to screening. Some respondents also identified discrimination and the fear of deportation as barriers.

*“Stigma, concerns that they may be deported once they access care . . .”*

*- Male, 45 years*

*“Stigma is a huge factor. The screening process appears to imply ‘contagiousness’ or considering the migrant ‘lesser’ or possessing “otherness” which is not aided by the physical premises of the screening activities frequently being poorly resourced, crowded, and often difficult to access.”*

*- Female, 54 years*

The participants also noted that migrants are a particular population with different needs. They are highly mobile, sometimes without documentation, and hard to reach, which limits their identification and can have a negative impact on screening coverage.

*“Poor documentation and mapping of the migrant population . . .”*

*- Male, 38 years*

Other potential barriers to screening identified by the respondents included misconceptions and lack of health literacy regarding active tuberculosis and infection.

*“Migrants from tuberculosis-endemic nations frequently lack understanding of LTBI conceptually, and it is time-consuming to incorporate this educational element into the process.”*

*- Female, 54 years*

Language and cultural barriers were reported to restrict access to health care. The experts also pointed out that the direct and indirect costs associated with migrants are significant barriers to tuberculosis screening. In addition, the costs associated with the screening in the health care provider perspective , as well as low numbers of and a lack of training of health care professionals, were identified as potential barriers to or limitations of tuberculosis screening. Two respondents mentioned the lack of an appropriate test for the diagnosis of infection, which increases the risk of overtreatment and of a higher number needed to treat, with the same risk of adverse effects from the medication.

*“Since the screening and diagnostic tests for LTBI are the same test, not including expert or good clinical assessment could risk overtreatment.”*

*- Female, 54 years*

Policy barriers were also identified by one participant who considered that tuberculosis in migrants is not a priority of the politicians in his country.

*“In Germany, there is as yet no comprehensive strategy to combat tuberculosis; quite low tuberculosis competence generally at public health care facilities, clinics, hospitals, and general practitioner offices [. . .] and money, the problem seems too small for most politicians.”*

*- Male, 57 years*

Table 4 illustrates the public health implications of the data derived from our thematic analysis.

**Table 4:** Public health implications derived from the thematic analysis.

Aspect	Key findings	Public health approach suggestion
Support for migrants	Nearly half of the participants (45.4%) emphasized the need for free access to tuberculosis health care services and treatment, for active and latent tuberculosis.	Implement policies for free access to tuberculosis health care services and treatment for all migrants, for active and latent tuberculosis.
Community involvement	The involvement of the community is crucial in supporting migrants with tuberculosis. It enhances engagement in health care processes and helps develop health-related behaviors and literacy.	Develop community-based health programs to engage migrants, leveraging local resources like pharmacies, clinics, and community centers.
Social support	Social support, including accommodation and food, is fundamental during the continuity of care. Tuberculosis screening should be part of a comprehensive health care package that includes social support tailored to the needs of the individual.	Integrate social support services, such as housing and food assistance, into tuberculosis care programs for migrants.
Person-centered care	Essential elements for continuity of care include person-centered approaches, health education, and literacy.	Focus on person-centered care models that include comprehensive health education and literacy programs tailored to the needs of migrants.
Use of technology	The use of VOT or DOT is important for ensuring adherence to treatment and follow-up.	Utilize VOT or DOT to monitor and support treatment adherence among migrants with tuberculosis, ensuring continuous and effective care.
Continuous follow-up	Continued care is necessary, with follow-up duration dependent on clinical conditions. Some experts suggest follow-up during the treatment period, while others recommend up to 12 months post-treatment for drug-resistant tuberculosis or 2 years in the case of preventive treatment refusal.	Establish follow-up protocols based on clinical conditions, including extended follow-up periods for drug-resistant tuberculosis and cases of preventive treatment refusal.
Screening practices	There is a consensus that tuberculosis screening should exclude active tuberculosis, especially in migrants from highly endemic countries. The screening should be adapted to the migrant characteristics and duration of stay. Short-term visitors from endemic areas should be screened for active tuberculosis using X-ray and sputum tests, whereas long-term entrants should also be screened for LTBI using an IGRA and QuantiFERON-TB Gold In-Tube assay.	Implement adaptive screening protocols based on the migrant’s stay duration and origin, utilizing X-ray, sputum tests, and IGRA for comprehensive tuberculosis detection.
Variability in practices	Screening practices vary by country, often including mandatory screening for active tuberculosis upon arrival and follow-up screenings for LTBI for long-term visitors. Some participants called for uniform guidelines across Europe to standardize practices.	Develop and adopt standardized tuberculosis screening guidelines across European countries to ensure consistent and effective screening practices.
Barriers and limitations	Stigma, discrimination, fear of deportation, and lack of understanding of tuberculosis were identified as major barriers. Other barriers include language and cultural differences, lack of documentation, high mobility of migrants, and costs associated with screening and treatment.	Implement culturally sensitive and accessible tuberculosis screening programs that address stigma, discrimination, and fear, providing support for undocumented and mobile populations.
Resource constraints	Participants highlighted the lack of training among health care professionals and inadequate resources for screening activities. There is also a concern about the lack of appropriate tests for LTBI diagnosis, leading to potential overtreatment and adverse effects.	Increase training for health care professionals in tuberculosis care and invest in resources for screening activities. Develop better diagnostic tools for LTBI to avoid overtreatment and manage adverse effects.
Policy and political will	Policy barriers exist, with some participants noting that tuberculosis in migrants is not prioritized by politicians in their countries. A comprehensive strategy and greater tuberculosis competence in public health services are needed.	Advocate for comprehensive national and international tuberculosis strategies that prioritize migrant health, supported by political commitment and sufficient funding.

VOT: video-observed therapy; DOT: directly observed therapy; IGRA: interferon-gamma release assay; and LTBI: latent tuberculosis infection.

## DISCUSSION

This study sought insights from tuberculosis experts on screening practices, revealing consensus on the lack of standardized guidelines and the benefits of harmonization. Experts agreed on the need for screening throughout the migrant journey, not just at the destination, and emphasized cross-country networks for continuity of care. Integrating community health services and involving social workers and trained nurses was highlighted as key to delivering people-centered care. Some low-incidence European countries now follow WHO and ECDC guidelines, screening high-risk migrants with chest X-rays for active tuberculosis and with TST or IGRA for LTBI. Although screening benefits public health by detecting cases of tuberculosis early, challenges remain in defining target populations, cost-effectiveness, and optimal test timing, as well as in addressing cultural and training barriers.^14^ ^-18^ As a result, screening procedures and best practices for detecting active tuberculosis and LTBI vary significantly across European countries. An essential strength of this qualitative study lies in its pioneering approach to current recommendations and perspectives of experts in tuberculosis from different countries regarding screening for LTBI and active tuberculosis. To our knowledge, this is the first study to provide a comprehensive overview of perspectives and recommendations on screening for tuberculosis. Including the perspectives of eleven experts who report on national recommendations, best practices, barriers to, and facilitators of screening enhances the utility of our findings. This qualitative study, involving experts in multiple countries, expands current evidence on tuberculosis screening. Respondents agreed that screening should occur throughout the migration process, not only at the destination, and that national networks are essential for continuity of care. They also unanimously emphasized the importance of providing free tuberculosis treatment for LTBI and active tuberculosis, together with access to primary care and health assessments to improve screening effectiveness. This study has limitations, including overrepresentation of the United Kingdom, a lack of representation of all WHO European Region countries and of low- and middle-income countries, a small sample size, and limited insights from migrants themselves, which restricts generalization and depth. Despite these limitations, the study offers unique strengths: it is, to our knowledge, the first to include a diverse range of tuberculosis experts across roles and countries; it used anonymized interviews for systematic data collection; and it provides a detailed dataset by including participant demographics and various aspects of tuberculosis screening. Considering all the limitations, barriers, and challenges highlighted by the participants, we proposed general and urgent strategies to increase the effectiveness of tuberculosis screening. Effective tuberculosis screening for migrants is facilitated by a network of primary care, public health, and hospitals, with community and social services integration playing key roles. Free access to screening, trained general practitioners, nursing staff, and social workers are priorities. Health literacy and promotion are essential, as are improving communication and considering the legal status and length of stay of migrants. Accurate testing and adapting to the diverse needs of migrants, together with clear, standardized guidelines, improve screening. Addressing resource constraints and ensuring long-term follow-up for LTBI are vital for better outcomes and harmonized procedures.

This study provides new insights into tuberculosis screening and treatment across different countries, particularly within the WHO European Region, and offers valuable guidance for developing harmonized guidelines for tuberculosis screening among migrants.
